# Impact of severe thrombocytopenia on the safety of splenectomy in patients with cirrhosis predominantly caused by Wilson's disease

**DOI:** 10.3389/fsurg.2026.1747136

**Published:** 2026-05-12

**Authors:** Huicong Min, Zhou Zheng, Qingsheng Yu, Yi Shen

**Affiliations:** 1 Anhui University of Chinese Medicine, Hefei, Anhui, China; 2 The Second Affiliated Hospital of Anhui University of Chinese Medicine, Hefei, Anhui, China; 3 The First Affiliated Hospital of Anhui University of Chinese Medicine, Hefei, Anhui, China

**Keywords:** cirrhosis, portal vein thrombosis, severe thrombocytopenia, splenectomy, surgical safety, Wilson’s disease

## Abstract

**Objective:**

Due to the risk of bleeding and other related complications, severe thrombocytopenia (PLT ≤ 30 × 10^9^/L) has been regarded as a relative contraindication for splenectomy in patients with liver cirrhosis. This study aimed to investigate the impact of severe thrombocytopenia on the safety of splenectomy in cirrhotic patients mainly caused by Wilson's disease.

**Methods:**

Patients were divided into three groups according to preoperative platelet count: mild, moderate, and severe thrombocytopenia. Baseline characteristics, surgical variables, postoperative outcomes, complications, and dynamic perioperative platelet counts were collected for all patients. Statistical analysis was performed using SPSS 27.0 to compare the differences in clinical data among the three groups.

**Results:**

No significant differences were observed among the three groups in baseline data, surgical indicators, mortality, gastric tube drainage volume, or total drainage volume (*P* > 0.05). The severe thrombocytopenia group had a significantly higher abdominal drainage volume at 48 h postoperatively than the mild group (*P* = 0.025), and a significantly higher incidence of postoperative portal vein thrombosis (*P* = 0.008, *P* = 0.010). There were significant differences in the incidence of postoperative fever among the three groups (*P* < 0.05), but no significant statistical difference was found between the severe group and the mild or moderate groups (*P* > 0.05). On postoperative days 3 and 7, the amylase level in abdominal drainage fluid of the severe group was significantly lower than that of the mild group (*P* = 0.005, *P* = 0.003). The postoperative platelet levels of all patients were significantly higher than the preoperative levels (*P* < 0.05); on postoperative days 1 and 7, there were significant differences in platelet levels between the severe thrombocytopenia group and the mild group (*P* < 0.05), but by postoperative day 7, the platelets of all three groups had recovered to the normal range.

**Conclusion:**

In Child-Pugh A/B patients with cirrhosis predominantly caused by Wilson's disease and hypersplenism, severe preoperative thrombocytopenia is not an absolute contraindication to splenectomy. Liver functional reserve is critical for surgical safety. However, severe thrombocytopenia is associated with an increased risk of PVT, which necessitates intensified perioperative anticoagulation.

## Introduction

Liver cirrhosis represents the end-stage manifestation chronic liver diseases, with portal hypertension (PHT) as the central complication (1). PHT leads to splenomegaly and hypersplenism, resulting in excessive destruction of platelets (PLT) and white blood cells (WBCs). Thrombocytopenia is the most common manifestation, which directly correlates with an increased risk of bleeding. Globally, cirrhosis ranks as the 13th leading causes of death, with approximately 683,300 cases reported in China, accounting for 14.9% of the global burden (2). Hypersplenism-induced thrombocytopenia not only escalates bleeding and infection risks but also may delay etiological treatment, such as copper chelation for Wilson's disease (WD) and antiviral therapy for viral hepatitis.

Splenectomy is an effective surgical intervention for hypersplenism, with well-documented benefits in alleviating thrombocytopenia, restoring hemostatic balance, and improving clinical outcomes (3–5). Clinical consensus supports the safety of splenectomy in patients with preoperative PLT counts >30 × 10^9^/L (6, 7), but the safety profile in those with PLT counts ≤30 × 10^9^/L remains controversial. Traditional views emphasize higher bleeding risk and recommend delayed surgery or alternative interventions such as splenic artery embolization. Therefore, clarifying the safety of splenectomy in patients with PLT counts ≤30 × 10^9^/L is of important clinical value for expanding surgical indications and improving patients' prognosis.

This retrospective study analyzed 115 patients with cirrhosis and hypersplenism dominated by WD who underwent splenectomy between January 2020 and February 2024, including 20 patients with severe thrombocytopenia (PLT counts ≤30 × 10^9^/L), to explore the feasibility and safety of splenectomy in these patients.

## Materials and methods

### Study subjects

This retrospective cohort study enrolled 115 patients with cirrhosis and hypersplenism who underwent elective splenectomy at our center between January 2020 and February 2024. All patients were diagnosed with cirrhosis and confirmed splenomegaly with hypersplenism.

### Splenectomy indications

(1) Persistent cytopenia unresponsive to pharmacological intervention, primarily affecting PLTs, erythrocytes, and leukocytes; (2) Hypersplenism interfering with etiological treatment of liver cirrhosis; (3) Enlarged spleen causing compression symptoms.

### Inclusion and exclusion criteria

Inclusion Criteria: (1) Cirrhotic patients who satisfied the diagnostic criteria for portal hypertension, exhibited splenomegaly and hypersplenism. (2) Patients who underwent splenectomy. (3) Patients of Child-Pugh class A or B. (4) Patients with complete preoperative and postoperative data, including complete blood counts, coagulation profiles, biochemical indices, and imaging findings.

Exclusion Criteria: (1) Severe coagulopathy or major organ dysfunction; (2) PLT transfusion within 1 week preoperatively; (3) Prior splenic artery embolization or TIPS; (4) Patients diagnosed with hepatocellular carcinoma, hepatic encephalopathy after admission, or in need of emergency surgery; (5) Preoperative liver function classified as Child-Pugh grade C.

### Grouping method

Based on the PLT count 1 day prior to surgery, patients were categorized into three groups (8, 9):

Mild Thrombocytopenia Group (Group A, *n* = 37): PLT > 50 × 10^9^/L;
Moderate Thrombocytopenia Group (Group B, *n* = 58): 30 < PLT ≤ 50 × 10^9^/L;
Severe Thrombocytopenia Group (Group C, *n* = 20): PLT ≤ 30 × 10^9^/L.

### Diagnostic criteria

#### Liver function classification by Child-Pugh

The Child-Pugh classification system was used to evaluate liver function (10). This classification was based on five factors: the stage of hepatic encephalopathy, degree of ascites, serum total bilirubin (TBIL), serum albumin (ALB), and prothrombin time (PT). Each factor was scored as 1, 2, or 3, depending on the severity, with a higher score indicating worse liver function.

#### Portal vein thrombosis (PVT)

The diagnosis of PVT was established on postoperative days 3, 7, and 14 based on Doppler ultrasound, contrast-enhanced computed tomography (CT), or magnetic resonance imaging (MRI) by detecting imaging evidence of thrombus in the main portal vein trunk and/or its intrahepatic branches, in accordance with established clinical practice guidelines (11, 12). Patients were classified as symptomatic or asymptomatic according to the presence of symptoms associated with portal vein obstruction.

Asymptomatic PVT was operationally defined as imaging-confirmed PVT in the absence of clinical manifestations, including abdominal pain, fever, nausea or vomiting, gastrointestinal bleeding, unexplained deterioration of liver function, or other signs suggestive of acute or subacute portal vein occlusion.

#### Postoperative intra-abdominal hemorrhage

Postoperative intra-abdominal hemorrhage should be suspected if any of the following criteria were fulfilled (13, 14): (1) A decrease in hemoglobin (Hb) level ≥ 30 g/L within 24–48 h postoperatively; (2) Requirement for blood transfusion support; (3) Persistently bloody drainage fluid or an abnormal increase in drainage volume (≥50 mL/h); (4) Radiological evidence of intra-abdominal hematoma or active bleeding.

### Pancreatic fistula

According to the ISGPS 2016 definition (15), a pancreatic fistula was diagnosed if the drainage amylase level exceeded three times the upper normal limit of serum amylase after the third postoperative day and required clinical intervention. If the amylase level was elevated without clinical symptoms, it was defined as a “biochemical leak” and was not included in the pancreatic fistula statistics.

### Postoperative fever

Postoperative fever was defined as a body temperature >38.5 °C within the first 3 postoperative days. Transfusion-related fever and drug-induced fever were excluded by retrospective review of transfusion records and medication history. Routine perioperative examinations, including complete blood count, inflammatory markers, drainage amylase and biochemical examinations, were used to exclude infectious and other pathological causes of fever.

### Perioperative management

#### Preoperative preparation

After comprehensive and targeted treatment, all patients had their liver function improved and stabilized at the Child-Pugh B level or above. One week before surgery, vitamin K1 was administered to correct or prevent coagulation dysfunction, thereby reducing the risk of bleeding. For patients with PT exceeding the normal control value by more than 3 s before surgery, prothrombin complex concentrate was infused preoperatively.

#### Surgical method

1. A left transrectus abdominal incision of approximately 18 cm was made, and the abdominal cavity was accessed layer by layer with a wound protector placed;
2. Exploration: The abdominal cavity was examined for the presence of ascites and the degree of splenic congestion and enlargement;
3. Main operative steps: The gastrocolic ligament was dissected to the right and extended to the gastrosplenic ligament. The gastrosplenic ligament was dissected, clamped, transected and ligated in bundles up to the superior pole of the spleen. The stomach was retracted to the right upper quadrant to expose the superior margin of the pancreas, and the splenic artery was dissected and ligated. The spleen was delivered out of the abdominal cavity. The splenophrenic and splenorenal ligaments were separated by a combination of blunt and sharp dissection, and the inferior pole of the spleen was mobilized. The splenorenal ligament was dissected, clamped, transected and ligated to achieve complete mobilization of the spleen. The splenic pedicle was clamped, transected, ligated and sutured in bundles, followed by splenectomy. The retroperitoneal splenic bed was sutured intermittently for hemostasis, and the wound surfaces of the gastric fundus and diaphragm were sutured and ligated to achieve hemostasis;
4. After confirming no active bleeding, a drainage tube was placed in the splenic fossa and exteriorized through a stab incision in the left upper quadrant, then fixed properly. The abdominal incision was closed layer by layer and covered with sterile dressings.

### Postoperative management

Postoperatively, patients continued receiving comprehensive treatment, including liver protection, maintenance of electrolyte balance, and nutritional support. Vital signs and changes in the nature and amount of abdominal drainage fluid were closely monitored, with timely removal of the drainage tube based on the drainage status. PLT and D-dimer concentrations were routinely monitored on postoperative days 7 and 14. In patients presenting with a preoperative hypercoagulable state, anticoagulant therapy with low-molecular-weight heparin or warfarin was initiated promptly after surgery and continued for 7–14 days. For patients diagnosed with early-stage PVT within 3 days postoperatively, immediate implementation of combined anticoagulation and thrombolytic therapy (e.g., urokinase) was clinically recommended. At the same time, patients were encouraged to get out of bed early to promote recovery.

### Study outcomes

The primary outcomes were perioperative surgical safety, including intraoperative blood loss, operation time, 14-day mortality and postoperative hospital stay. Secondary outcomes included PVT, drainage volume, postoperative fever, pancreatic fistula, postoperative bleeding and 14-day postoperative dynamic PLT recovery.

### Research indicators and data collection

#### Baseline characteristics

Age, gender, etiology of cirrhosis, comorbidities (hypertension, diabetes, anemia, previous abdominal surgery), smoking and drinking history, Child Pugh classification, liver stiffness measurement (LSM), aspartate minotransferase to PLT ratio index (APRI), fibrosis 4 score (FIB_4), portal hypertension, esophageal varices, hypertensive gastropathy and jaundice.

#### Primary outcomes

Operative duration, intraoperative blood loss, postoperative hospital stay and 14 day postoperative mortality.

#### Secondary outcomes

Postoperative complications, including postoperative bleeding, fever, PVT, pancreatic fistula the difference in Hb between postoperative day 3 and preoperative baseline (Δ Hb) and PLT counts (preoperative and postoperative days 1, 7, 14).

#### Postoperative drainage volumes

Forty-eight hours postoperative nasogastric tube drainage volume, abdominal drainage volume, and total drainage volume, and amylase levels in abdominal drainage fluid.

### Statistical methods

SPSS 27.0 software was used for data analysis. For continuous variables, normality was assessed using the Shapiro–Wilk test (or Kolmogorov–Smirnov test). Normally distributed variables were presented as mean ± standard deviation (*x¯ ± s*) and compared between groups using the independent *t*-test. Non-normally distributed variables were presented as median (P25–P75) [M (P25–P75)], and between-group comparisons were performed using the Kruskal–Wallis *H* test; pairwise comparisons were conducted with the Mann–Whitney *U* test. Categorical variables were presented as counts and percentages (*n* %). Between-group comparisons for categorical variables were performed using the Pearson's *χ*^2^ test when the expected frequency in each cell was ≥5, or Fisher's exact test when any expected frequency was <5. A *P*-value <0.05 was considered statistically significant. The significance level for all tests was set at *α* = 0.05.

## Results

### Comparison of baseline data

Among all patients, 58 were male (50.4%) and 57 were female (49.6%), with an average age of 34.39 ± 14.83 years. The etiology of cirrhosis was predominantly WD (98 cases, 85.2%), with the remaining cases being viral hepatitis-related cirrhosis or alcoholic cirrhosis (17 cases, 14.8%). Preoperative liver function classification by Child-Pugh showed 88 patients (76.5%) were classified as Child-Pugh A and 27 patients (23.5%) as Child-Pugh B. There were no statistically significant differences in gender, age, etiology of cirrhosis, medical history (hypertension, diabetes, abdominal surgery history, anemia), personal history (smoking, drinking), Child-Pugh classification, oesophageal varices, hypertensive gastropathy, jaunfice, LSM, APRI, FIB4 among the three groups (*P* > 0.05). Thus, baseline characteristics were well-balanced and did not confound the between-group comparisons (Table 1).

**Table 1:** Comparison of baseline characteristics among three groups [*x¯* ± *s* M (P25–P75) or *n* (%)].

Group	Group A (*n* = 37)	Group B (*n* = 58)	Group C (*n* = 20)	*P*
Gender				0.642
Male	18 (48.6)	28 (48.3)	12 (60.0)	
Female	19 (51.4)	30 (51.7)	8 (40.0)	
Age (years)	33.00 (27.00–49.00)	28.00 (22.00–38.00)	29.50 (21.50–43.00)	0.218
Cause of cirrhosis				0.778
HLD cirrhosis (cases)	32 (86.5)	50 (86.2)	16 (80.0)	
Others (cases)	5 (13.5)	8 (13.8)	4 (20.0)	
History of hypertension				0.232
No (cases)	37 (100.0)	54 (93.1)	20 (100.0)	
Yes (cases)	0 (0.0)	4 (6.9)	0 (0.0)	
History of diabetes				0.708
No (cases)	35 (94.6)	55 (94.8)	20 (100.0)	
Yes (cases)	2 (5.4)	3 (5.2)	0 (0.0)	
History of abdominal surgery				1.000
No (cases)	28 (75.7)	44 (75.9)	15 (75.0)	
Yes (cases)	9 (24.3)	14 (24.1)	5 (25.0)	
History of anemia				0.408
No (cases)	12 (32.4)	21 (36.2)	4 (20.0)	
Yes (cases)	25 (67.6)	37 (63.8)	16 (80.0)	
Smoking history				0.785
No (cases)	35 (94.6)	54 (93.1)	18 (90.0)	
Yes (cases)	2 (5.4)	4 (6.9)	2 (10.0)	
Alcohol consumption history				0.642
No (cases)	34 (91.9)	56 (96.6)	19 (95.0)	
Yes (cases)	3 (8.1)	2 (3.4)	1 (5.0)	
Child-Pugh classification				0.641
A (cases)	30 (81.1)	44 (75.9)	14 (70.0)	
B (cases)	7 (18.9)	14 (24.1)	6 (30.0)	
Oesophageal varices				0.447
No (cases)	24 (64.9)	30 (51.7)	11 (55.0)	
Yes (cases)	13 (35.1)	28 (48.3)	9 (45.0)	
Hypertensive gastropathy				0.064
No (cases)	34 (91.9)	44 (75.9)	14 (70.0)	
Yes (cases)	3 (8.1)	14 (24.1)	6 (30.0)	
Jaunfice				0.449
No (cases)	23 (62.2)	42 (72.4)	12 (60.0)	
Yes (cases)	14 (37.8)	16 (27.6)	8 (40.0)	
LSM (kPa)	19.20 (16.95–21.30)	20.00 (16.53–22.33)	19.60 (17.05–22.35)	0.825
APRI	2.35 (2.05–2.67)	2.16 (1.89–2.46)	2.31 (1.85–2.72)	0.118
FIB4	4.70 (3.95–5.35)	4.80 (3.95–5.83)	5.32 (4.68–5.75)	0.066

(1) Group A: mild thrombocytopenia group; Group B: moderate thrombocytopenia group; Group C: severe thrombocytopenia group. (2) Others: hepatitis-related cirrhosis, alcoholic cirrhosis.

### Primary endpoints

No clinically significant differences were observed among the three groups in operative duration, intraoperative blood loss, or the length of hospital stay (*P* > 0.05). No deaths occurred within 14 days postoperatively in any of the three groups. Postoperative drainage observation showed no obvious bloody drainage in all groups; only the severe thrombocytopenia group had a significantly higher abdominal drainage volume at 48 h postoperatively than the mild thrombocytopenia group (*P* = 0.009). However, the abdominal drainage volume in all three groups decreased to below 50 mL from the 3rd postoperative day. The postoperative Hb level exhibited an overall upward trend in all three groups, and no case of Hb decrease exceeding 30 g/L was identified. Meanwhile, routine postoperative abdominal imaging reexamination revealed no obvious intra-abdominal effusion or hematoma. Based on the characteristics of drainage fluid, abdominal imaging findings and changes in Hb, no obvious overt bleeding was detected postoperatively in any group. Since qualitative analysis of the drainage fluid was not performed, the possibility of occult bleeding could not be completely excluded (Table 2).

**Table 2:** Intraoperative and 48-hour postoperative drainage and recovery parameters [*x¯ ± s* or M (P25–P75)].

Clinical indicators	Group A (*n* = 37)	Group B (*n* = 58)	Group C (*n* = 20)	*P*	*Pa*	*Pb*
Surgery duration (min)	146.00 (118.00–180.00)	155.00 (130.00–182.00)	147.00 (128.50–186.00)	0.619		
Intraoperative blood loss (mL)	100.00 (50.00–200.00)	175.00 (100.00–200.00)	200.00 (55.00–350.00)	0.221		
48 h postoperatively						
Gastric tube drainage volume (mL)	20.00 (0.00–100.00)	8.50 (0.00–100.00)	40.00 (0.00–140.00)	0.595		
Abdominal drainage volume (mL)	235.00 (100.00–320.00)	248.50 (142.50–400.00)	370.00 (205.00–645.00)	0.030	0.025	0.227
Total drainage volume (mL)	300.00 (165.00–455.00)	300.00 (169.50–537.50)	462.50 (252.50–720.00)	0.112		
Δ Hb (g/L)	6.00 (5.00–7.50)	7.00 (5.50–9.00)	6.00 (5.00–8.00)	0.176		
Postoperative hospital stay (days)	15.00 (14.00–18.00)	16.00 (14.00–21.00)	16.50 (14.50–20.50)	0.361		

(1) Group A: mild thrombocytopenia group; Group B: moderate thrombocytopenia group; Group C: severe thrombocytopenia group. (2) *P* indicates comparisons among the three groups; *Pa* represents comparison between Group C and Group A; *Pb* represents comparison between Group C and Group B.

### Secondary endpoints

#### Postoperative PVT

**Figure 1:**
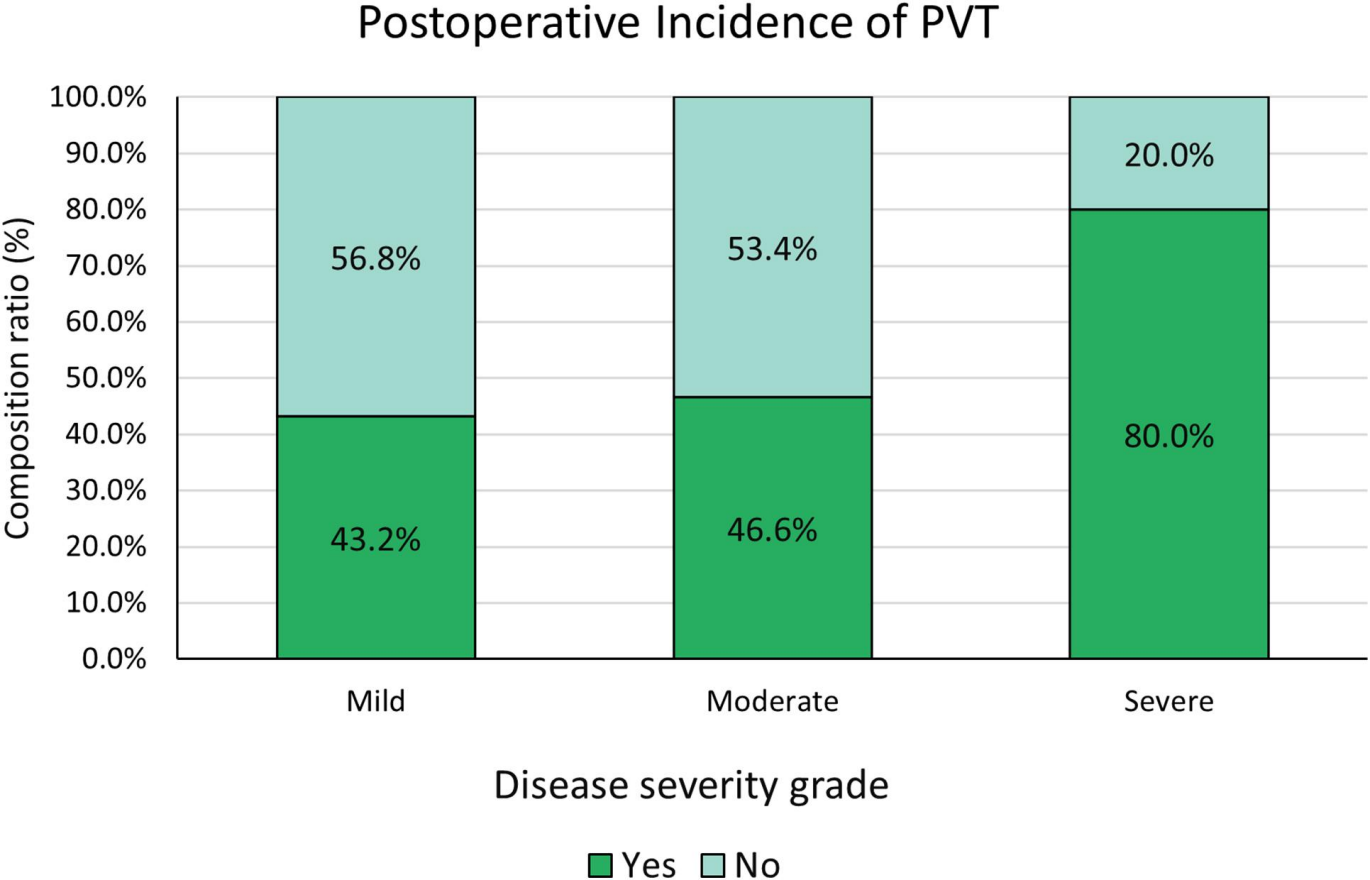
Comparison of perioperative PVT incidence among three groups. (1) Yes: postoperative PVT occurred; (2) No: No postoperative PVT occurred.

The incidence of PVT in the mild group was 43.2% (16/37), 46.6% (27/58) in the moderate group, and in the severe group was 80.0% (16/20). There was a statistically significant difference among the three groups (*Pa* = 0.008, *Pb* = 0.010) (Figure 1). All PVT events were asymptomatic and resolved with anticoagulation (Table 3).

**Table 3:** Incidence of perioperative complications among three groups [*x¯ ± s*, M (P25–P75) or (*n* %)].

Clinical indicators	Group A (*n* = 37)	Group B (*n* = 58)	Group C (*n* = 20)	*P*	*Pa*	*Pb*
Postoperative PVT				0.018	0.008	0.010
No (cases)	21 (56.8)	31 (53.4)	4 (20.0)			
Yes (cases)	16 (43.2)	27 (46.6)	16 (80.0)			
Postoperative fever				0.029	0.233	0.459
No (cases)	13 (35.1)	7 (12.1)	4 (20.0)			
Yes (cases)	24 (64.9)	51 (87.9)	16 (80.0)			
Amylase levels						
Postoperative D3 (U/mL)	316.00 (100.00–1,145.00)	167.00 (64.00–446.00)	76.00 (46.00–189.00)	0.007	0.005	0.111
Postoperative D7 (U/mL)	73.00 (37.00–165.00)	53.00 (30.50–89.00)	37.00 (22.00–55.00)	0.003	0.003	0.314

(1) Group A: mild thrombocytopenia group; Group B: moderate thrombocytopenia group; Group C: severe thrombocytopenia group. (2) *P* indicates comparisons among the three groups; *Pa* represents comparison between Group C and Group A; *Pb* represents comparison between Group C and Group B.

#### Postoperative fever

The incidence of postoperative fever in the mild group was 64.9% (24/37), in the moderate group was 87.9% (51/58), and in the severe group was 80.0% (16/20). The overall chi-square test indicated a significant difference among groups (*χ*^2^ = 7.024, *P* = 0.029), suggesting an underlying difference in postoperative fever rates across thrombocytopenia strata. *post-hoc* pairwise comparisons showed no significant differences between group C and groups A/B (*Pa* = 0.233, *Pb* = 0.459), which was most likely due to insufficient statistical power caused by the small sample size of the severe thrombocytopenia group rather than a true absence of association (Table 3).

#### Postoperative pancreatic fistula

The median amylase levels in abdominal drainage fluid on postoperative days 3 and 7 were compared among the three groups: Mild group: Postoperative day 3, 316.00 U/L; day 7, 73.00 U/L; Moderate group: Postoperative day 3, 167.00 U/L; day 7, 53.00 U/L; Severe group: Postoperative day 3, 76.00 U/L; day 7, 37.00 U/L. There were no significant differences in amylase levels between the severe and moderate groups on postoperative days 3 (*P* = 0.116) and 7 (*P* = 0.510). However, there were significant differences between the severe and mild groups on postoperative days 3 (*P* = 0.005) and 7 (*P* = 0.010). No clinical pancreatic fistula was observed across all groups (Table 3).

### Dynamic PLT changes

**Figure 2:**
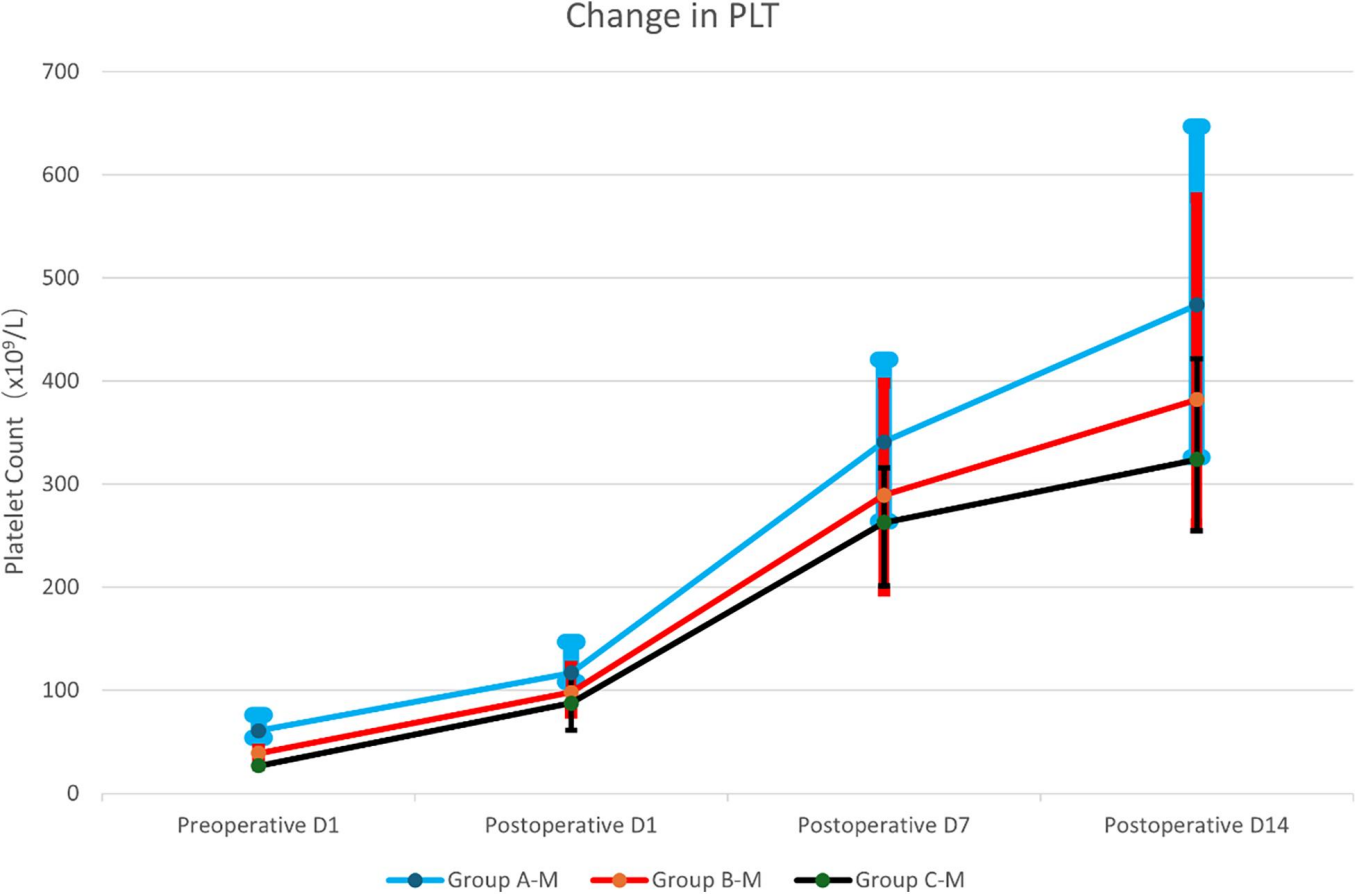
Dynamic changes in perioperative PLT counts. (1) Group A: mild thrombocytopenia group; Group B: moderate thrombocytopenia group; Group C: severe thrombocytopenia group. (2) M, median.

There was a significant significant overall difference in preoperative PLT levels among the three groups (*P* < 0.001). The PLT counts of the three groups on postoperative days 1, 7, and 14 were all higher than the preoperative values, showing a continuous increasing trend overall. Significant overall intergroup differences were observed on postoperative days 1 and 7 (*P* < 0.05), and pairwise comparison only revealed a statistically significant difference between the mild and severe groups on postoperative day 1 (*P* < 0.001). By postoperative day 14, there was no statistically significant intergroup difference in PLT counts among the three groups (*P* > 0.05), suggesting that the overall trend of postoperative PLT recovery was basically consistent in patients with different degrees of thrombocytopenia (Table 4 and Figure 2).

**Table 4:** Comparison of dynamic changes in perioperative PLT counts [(×10^9^/L), *x¯ ± s* or M (P25–P75)].

Time	Group A (*n* = 37)	Group B (*n* = 58)	Group C (*n* = 20)	*P*	*Pa*	*Pb*
Preoperative D1	61.00 (54.00–76.00)	39.00 (35.00–43.00)	27.00 (24.00–28.00)	<0.001	<0.001	<0.001
Postoperative D1	117.00 (108.00–147.00)	98.00 (78.00–123.00)	87.50 (61.50–116.00)	<0.001	<0.001	0.989
Postoperative D7	341.00 (264.00–421.00)	289.50 (196.00–398.00)	263.00 (201.50–316.00)	0.038	0.090	1.000
Postoperative D14	474.00 (326.00–647.00)	382.00 (261.50–578.00)	324.00 (255.00–422.00)	0.100		

(1) Group A: mild thrombocytopenia group; Group B: moderate thrombocytopenia group; Group C: severe thrombocytopenia group. (2) *P* indicates comparisons among the three groups; *Pa* represents comparison between Group C and Group A; *Pb* represents comparison between Group C and Group B.

## Discussion

This study aimed to investigate the impact of severe thrombocytopenia (PLT counts ≤ 30 × 10^9^/L) on the surgical safety of splenectomy in patients with Child-Pugh class A/B cirrhosis and hypersplenism predominantly caused by WD. The core finding was that severe thrombocytopenia was not associated with increased risks of intraoperative bleeding, postoperative bleeding, mortality, or prolonged hospitalization. These observations suggest that severe thrombocytopenia may not be an absolute contraindication to splenectomy in Child-Pugh class A/B patients. Meanwhile, PLT levels in all three groups returned to the normal range on postoperative day 7. However, severe thrombocytopenia was associated with increased 48-h abdominal drainage volume and the incidence of asymptomatic PVT.

Traditional clinical views hold that a PLT count >50 × 10^9^/L is the safe threshold for splenectomy, and patients with this count have a low risk of postoperative complications (9, 16, 17). In contrast, severe thrombocytopenia with a PLT count ≤30 × 10^9^/L is considered to significantly increase the risk of intraoperative and postoperative bleeding, and the procedure's surgical safety has long been controversial (18). The results of this study are inconsistent with the above traditional perceptions. Under standardized perioperative management, there were no significant differences in operative time and intraoperative blood loss between the severe thrombocytopenia group and the mild and moderate thrombocytopenia groups, and no perioperative death or severe bleeding complications occurred in all subjects, suggesting that severe thrombocytopenia does not significantly increase the risk of bleeding associated with splenectomy. In terms of dynamic changes in PLT counts, although there were statistically significant differences in baseline PLT levels among the three groups before surgery, PLT counts in the severe thrombocytopenia group recovered rapidly after surgery, which were only significantly lower than those in the mild group on postoperative day 1. By postoperative day 7, there were no statistically significant differences in PLT levels among the three groups, and all returned to the normal range, suggesting that patients with severe thrombocytopenia have good hematopoietic recovery potential after surgery.

From the perspective of pathophysiological mechanisms, the above clinical phenomena are closely related to PLT functional compensation and preserved hepatic synthetic function. Thrombocytopenia in patients with liver cirrhosis and splenomegaly is mainly caused by excessive splenic destruction. Patients have sufficient reserves of PLT precursors in the bone marrow (19), and most residual PLTs in the body are in an activated state (20). Therefore, even if the peripheral blood PLT count is low, the body's hemostatic function can still be effectively compensated. Meanwhile, the pathological destruction of PLTs is significantly reduced after splenectomy, and the number of functionally normal PLTs increases, which also reasonably explains why patients in the severe thrombocytopenia group had low preoperative PLT levels without bleeding complications. In addition, this study strictly excluded patients with Child-Pugh grade C liver dysfunction and only enrolled patients with Child-Pugh grade A/B. Existing studies have confirmed that the Child-Pugh classification, rather than PLT count alone, is an independent risk factor for bleeding after splenectomy (8). Therefore, well-preserved liver function is one of the important prerequisites for reducing the incidence of complications following splenectomy in the present study.

Notably, patients with severe thrombocytopenia had a significantly higher risk of postoperative PVT, the most prominent perioperative adverse event in this study. Pathophysiologically, PHT causes splenic congestion and increased PLT sequestration, while bone marrow thrombopoiesis remains intact. Splenectomy thus induces a more pronounced PLT rebound, leading to a hypercoagulable state in the portal venous system. Although all PVT cases were asymptomatic, asymptomatic thrombosis is not benign and may progress to portal vein occlusion, intestinal ischemia, and impaired hepatic perfusion without intervention. Therefore, severe preoperative thrombocytopenia is a risk factor for PVT, requiring close monitoring and individualized anticoagulation to improve perioperative safety.

The abdominal drainage volume at 48 h postoperatively was significantly higher in the severe thrombocytopenia group than in the mild thrombocytopenia group (*P* = 0.025). This finding may be attributed to increased peritoneal exudation caused by PHT in patients with cirrhosis (21). As this was a retrospective study, routine component analysis of drainage fluid was not performed, so occult bleeding components could not be quantitatively excluded. However, based on the characteristics of drainage fluid, abdominal imaging findings and changes in hemoglobin, no obvious overt bleeding occurred postoperatively in any of the three groups. Moreover, the abdominal drainage volume decreased to a low level within 3 days after surgery, and no severe bleeding-related complications were observed. Nevertheless, this significant intergroup difference indicates increased peritoneal exudation in patients with severe thrombocytopenia, which still warrants close clinical attention.

Differences were observed among the three groups in postoperative fever and amylase levels in abdominal drainage fluid. The overall incidence of postoperative fever showed a statistically significant difference among groups, but pairwise comparisons did not reach statistical significance, which was limited by the small sample size of the severe thrombocytopenia group. Therefore, the potential association between the severity of thrombocytopenia and postoperative fever cannot be excluded; transient fever in this cohort was more likely related to surgical trauma rather than thrombocytopenia alone. On postoperative days 3 and 7, the amylase level in abdominal drainage fluid was lower in the severe thrombocytopenia group than in the mild thrombocytopenia group; however, no clinical pancreatic fistula occurred in any group, indicating that this difference had limited clinical significance.

Based on the results of this study, we suggest that for cirrhosis patients with splenomegaly, if the liver function is classified as Child-Pugh A/B, even with a PLT count ≤30 × 10^9^/L, splenectomy may be considered in patients with adequate liver function, accompanied by strict perioperative anticoagulation management. Furthermore, in perioperative management, active correction of coagulation is recommended, and routine PLT transfusion is not advocated. Early postoperative anticoagulation with low molecular weight heparin may be considered to reduce the risk of PVT. From a clinical perspective, the decision to perform splenectomy in patients with severe thrombocytopenia should not be based solely on PLT count, but rather on a comprehensive assessment of bleeding risk, thrombotic risk, and liver function reserve. Meanwhile, the above results indicate that meticulous dissection and adequate protection of the pancreatic tail during splenectomy can effectively reduce the risk of postoperative pancreas-related complications.

In addition, 85.2% of the study population consisted of patients with WD. In contrast to viral or alcoholic cirrhosis, which is frequently associated with markedly reduced levels of multiple coagulation factors, enhanced fibrinolytic activity, and a significant bleeding tendency due to chronic inflammation, progressive hepatocellular necrosis, and severe hepatic synthetic dysfunction (22–24), WD is characterized by hepatocellular injury mainly mediated by copper toxicity rather than persistent inflammatory necrosis (25). Consequently, patients with WD exhibit relatively preserved hepatic synthetic function and milder disturbances in the coagulation cascade even in the presence of thrombocytopenia secondary to hypersplenism. In comparison, viral and alcoholic cirrhosis more commonly present with combined deficiencies of procoagulant and anticoagulant factors, leading to a more significant imbalance in hemostasis. Nevertheless, all patients in this study were classified as Child–Pugh class A or B with compensated liver function, and the central mechanism underlying thrombocytopenia was hypersplenism rather than global hepatic failure.

This study has several limitations. First, the single-center retrospective design entails potential selection bias. Second, the relatively small sample size of the severe thrombocytopenia subgroup provides insufficient statistical power and limited evidence for clinical safety; thus, future multicenter and large-sample studies are warranted to verify safety and minimize statistical bias. Third, the predominance of WD restricts the generalizability of the present findings to other etiological subtypes of liver cirrhosis. Furthermore, the follow-up duration was relatively brief and inadequate for assessing long-term outcomes, particularly PVT. Finally, given the limited evidence specific to WD, conventional PLT cutoff values were adopted in this study, which may not be optimally suitable for this particular patient cohort.

## Conclusion

In Child-Pugh A/B patients with cirrhosis predominantly caused by WD and hypersplenism, a PLT count ≤30 × 10^9^/L is not an absolute contraindication for splenectomy and does not significantly increase the risk of bleeding. Liver functional reserve is the primary determinant of perioperative outcomes. However, severe thrombocytopenia is a risk factor for PVT, which requires close monitoring and individualized anticoagulation to improve perioperative safety.

## Data Availability

The raw data supporting the conclusions of this article will be made available by the authors, without undue reservation.
